# The WEVO Technique: A Novel Dual-Device Endovascular Strategy for Non-Geometric Wide Neck Bifurcation Aneurysms

**DOI:** 10.1007/s00062-026-01640-w

**Published:** 2026-03-24

**Authors:** Sarah J. Snyder, Om H. Gandhi, Sami Almasri, Jaeha Kim, Shirley Yuan, Nathan Yu, Aaron Anandarajah, Suehyb G. Alkhatib, Sandeep Kandregula, Linda Bagley, Omar A. Choudhri

**Affiliations:** 1 Lewis Katz School of Medicine, Temple University, Philadelphia, United States; 2 Department of Neurosurgery, University of Pennsylvania, Philadelphia, United States; 3 Radiology Associates of Richmond, Richmond, United States; 4 Department of Radiology, University of Pennsylvania, Philadelphia, United States; 5 Department of Neurosurgery, University of Pennsylvania, Philadelphia, United States

**Keywords:** Cerebral aneurysm, Non-geometric aneurysm, Bifurcation aneurysm, Endovascular techniques, Stenting, Intrasaccular device, Novel technique

## Abstract

**Purpose:**

Non-geometric wide-neck bifurcation aneurysms (WNBAs) present with treatment challenges of unfavorable neck morphology at bifurcation sites combined with irregular saccular geometry. While microsurgical clipping remains the traditional standard treatment, unsuitable candidates often require endovascular alternatives. We developed the WEVO technique, combining a volumetrically matched Woven EndoBridge (WEB) placement with a LVIS EVO braided stent deployment to achieve device stabilization and neck reconstruction with enhanced metal coverage.

**Methods:**

We conducted a retrospective review of 21 patients treated for non-geometric WNBAs. Patient demographics, aneurysm characteristics, procedural parameters, and clinical outcomes were analyzed. The LVIS EVO stent was deployed using a “shouldering” technique to compress stent cells across the aneurysm neck. All patients received dual antiplatelet therapy. Aneurysm occlusion was assessed using the WEB Occlusion Scale (WOS) at 3 to 6 months and at 12 months.

**Results:**

Median age was 71 (IQR: 64–76); 71% were female. All aneurysms were unruptured with median volume 81.4 mm^3^ (IQR: 46.0-147.1). Most common locations included middle cerebral artery (33%) and basilar artery (33%). Upper extremity arterial access was used in all cases. Immediate contrast stasis was achieved in all patients. One patient developed transient intra-procedural stent thrombus that resolved with intra-arterial tirofiban. No other complications occurred. At 3 to 6‑month follow-up (*n* = 16), adequate occlusion (WOS Grade A or AB) was achieved in 87%. At 12-month follow-up (*n* = 11), adequate occlusion was maintained in all patients.

**Conclusion:**

The WEVO technique provides a safe and effective endovascular treatment option for non-geometric WNBAs by combining intrasaccular flow disruption with neck scaffolding and increased metal coverage.

**Supplementary Information:**

The online version of this article (https://doi.org/10.1007/s00062-026-01640-w) contains supplementary material, which is available to authorized users.

## Introduction

Wide-neck aneurysms are defined as aneurysms with a neck width greater than 4 mm or a dome-to-neck ratio less than 2, [1] and are considered wide-neck bifurcation aneurysms (WNBAs) when arising at arterial bifurcations. These aneurysms account for 26–36% of all cerebral aneurysms and occur most commonly at the middle cerebral artery (MCA) bifurcation and basilar artery apex [2]. Endovascular treatment of WNBAs remains challenging due to high recurrence rates and the difficulty achieving complete aneurysm occlusion without compromising parent vessel patency [3].

Multiple endovascular strategies have been employed for these aneurysms, including stent-assisted coiling with laser-cut stents (Neuroform Atlas, Enterprise), [4–8] stent-assisted coiling with braided stents (LVIS, Leo), [9] neck reconstruction devices (PulseRider, eCLIPs), [10, 11] as well as intrasaccular devices including the Woven EndoBridge (WEB), [2] Contour Neurovascular System, [12] and ARTISSE [13, 14]. One of the primary advantages of intrasaccular devices is the lack of need for a stent [15]. This allows omission of dual antiplatelet therapy and mitigates risks of in-stent thrombosis and stenosis, [15] making intrasaccular devices valuable in acute ruptured settings. Among intrasaccular options, the WEB is the most established having been extensively evaluated by multiple prospective, multicenter studies, demonstrating occlusion rates comparable to stent-assisted coiling with slightly lower complication and retreatment rates [16–18].

WNBAs with non-geometric morphology, characterized by non-spherical and non-cylindrical irregular shapes and daughter lobules, present a fundamental treatment challenge. Standard WEB device sizing algorithms rely on width-based measurements assuming relatively spherical or ellipsoidal geometry while others utilize aligning aneurysm and WEB device volumes [19, 20]. In non-geometric aneurysms, these algorithms become unreliable, resulting in unpredictable volume matching that compromises both adequate wall apposition and volumetric filling of the sac [2, 21]. These complex morphologies have traditionally required microsurgical neck reconstruction with titanium clips. While WEB device therapy offers an endovascular alternative for patients who cannot tolerate craniotomy, standalone intrasaccular treatment often represents a compromise solution with significantly lower rates of complete aneurysm occlusion compared to clipping [1].

To address these limitations, we developed a hybrid technique combining intrasaccular and endoluminal approaches. The technique involves sequential placement of a WEB device followed by deployment of an LVIS EVO braided stent [22] from the proximal parent artery through one of the bifurcating branches. The LVIS EVO stent serves dual purposes: it provides structural scaffolding to contain WEB device protrusion into the parent vessel, and its high mesh density (up to 28% metal coverage) [22] increases metal coverage at the aneurysm neck, enhancing flow disruption and promoting intrasaccular thrombosis. We term this combined approach the WEVO technique (WEB plus LVIS EVO). In this study, we evaluate the safety and efficacy of the WEVO technique for the treatment of non-geometric WNBAs.

## Methods

### Study Design and Patient Selection

We conducted a retrospective cohort study of patients with intracranial aneurysms who underwent endovascular treatment between January 2024 and July 2025. The Institutional Review Board waived the requirement for informed consent due to the retrospective nature of this study. Patients were eligible for inclusion if they presented with a non-geometric WNBA and underwent endovascular treatment using a combined approach with a WEB device (Terumo Neuro, Aliso Viejo, California, USA) and LVIS EVO stent (Terumo Neuro, Aliso Viejo, California, USA) deployed across the aneurysm neck using the shouldering technique.

### Endovascular Treatment Technique

All procedures utilized a dual-device strategy combining WEB device implantation with LVIS EVO stent deployment. Vascular access was obtained via the radial or ulnar artery based on operator preference, patient anatomy, and the dominant vessel. A guide catheter was advanced into the cervical target vessel, and an intermediate catheter was navigated distally to provide stable platform support. The aneurysm was then selected using a VIA microcatheter (Terumo Neuro, Aliso Viejo, California, USA) for WEB device deployment.

WEB device sizing followed established clinical recommendations with deliberate lateral oversizing by 1 to 2 mm to ensure adequate width compression at the aneurysm neck. Volumetric matching with minimal oversizing was employed to achieve adequate filling of the non-geometric aneurysm sac while avoiding excessive device compression. Aneurysm measurements were completed on magnified oblique working projection images using height, width, and neck measurements. Aneurysm volumes were calculated based on 3D angiographic images and compared to a standardized WEB device volume chart. Following WEB device deployment, a microcatheter for stent delivery was navigated into the appropriate branch vessel. The shouldering technique was defined as intentional application of forward pressure on the stent delivery system during LVIS EVO deployment, resulting in progressive packing and compression of stent cells across the aneurysm neck.

**Fig. 1:**
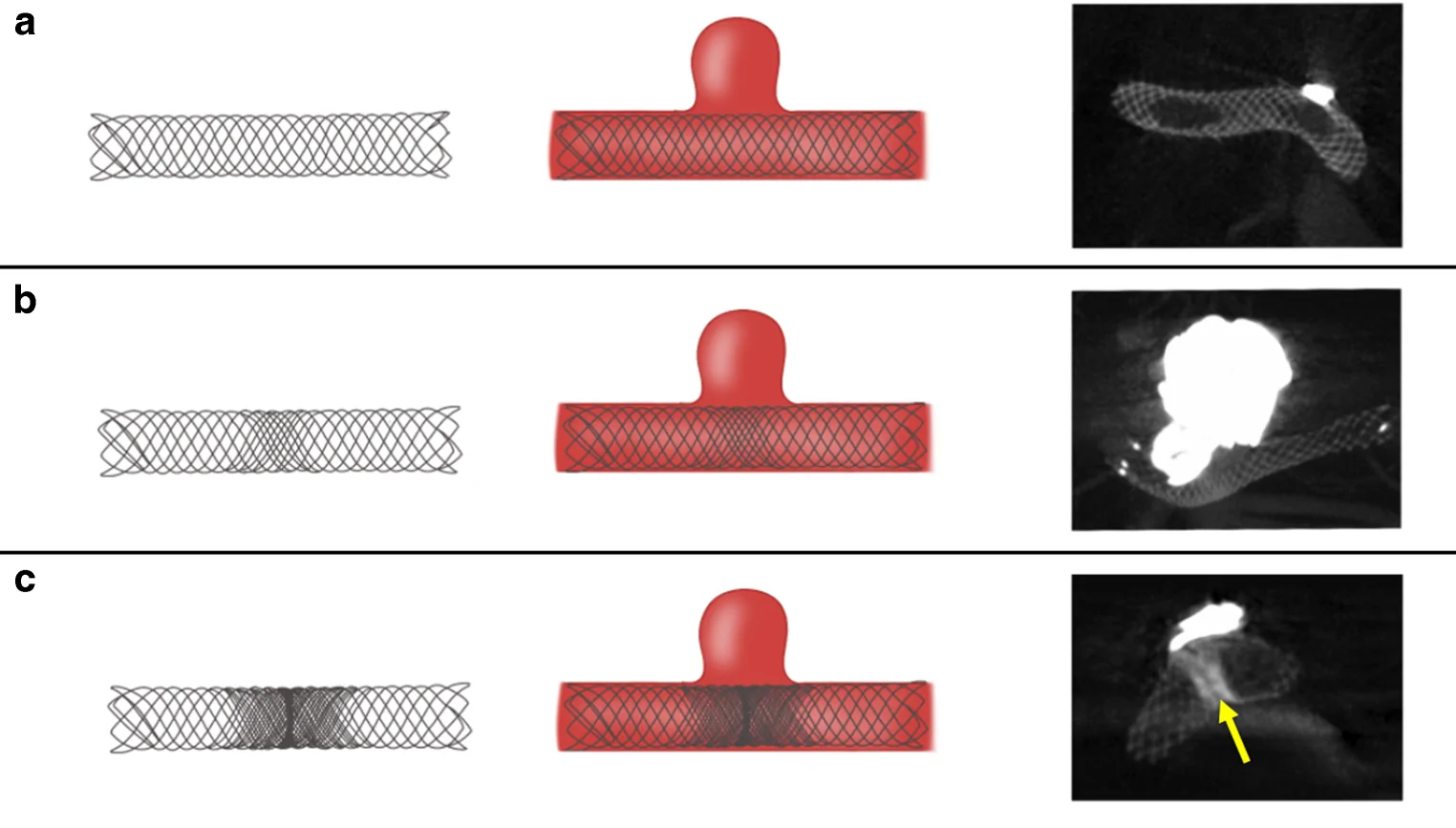
Illustration of the shouldering technique demonstrating progressive stent cell constriction across the aneurysm neck, shown schematically as the stent alone (left), across the aneurysm neck (center), and corresponding radiographic images (right). **a** Minimal shouldering. **b** Moderate shouldering. **c** Maximal shouldering with stent cell collapse, characterized angiographically by the “ring sign.” (arrow)

Immediately following stent deployment, a 14-second microDyna CT imaging study (Siemens Healthineers, Erlangen, Germany) was performed to assess the degree of shouldering and device packing across the aneurysm neck. The extent of shouldering was graded on a three-level scale based on the extent of stent compression at the aneurysm neck (Fig. 1).

All patients received dual antiplatelet therapy for three months according to institutional protocols for stent-assisted aneurysm treatment. Platelet function testing using VerifyNow (Werfen, Bedford, MA) P2Y12 reaction unit assays was performed to confirm therapeutic antiplatelet response before the procedure.

### Data Collection, Outcome Assessment, and Analysis

**Fig. 2:**
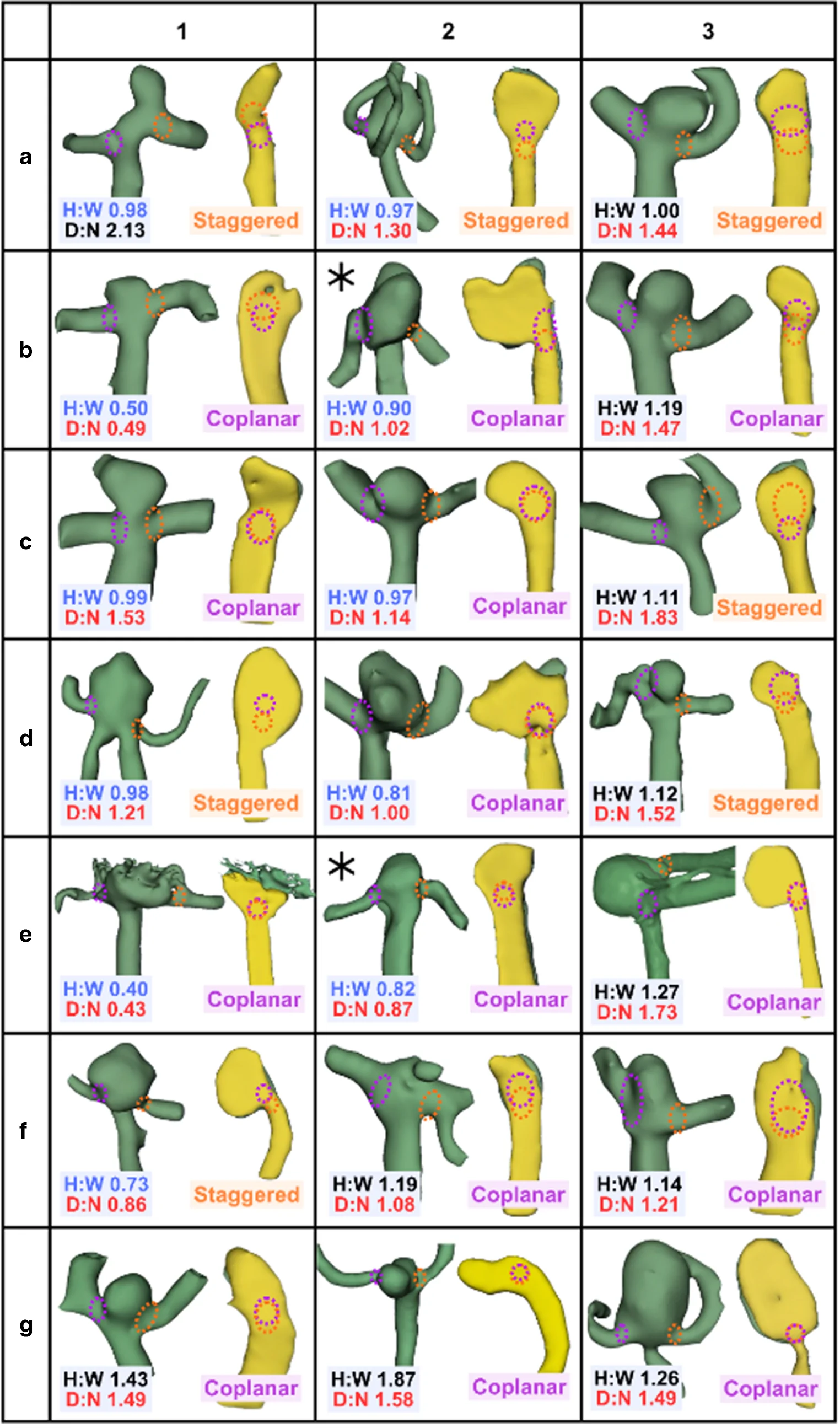
3D reconstructions (green) and corresponding cross-sections (yellow) of the 21 aneurysms included in this study. Classification was categorized based on height-to-width (H:W) ratio, dome-to-neck (D:N) ratio, and branching pattern. Pancake/shallow morphology was defined as H:W < 1, characterized by broad-based, flat lesions (blue). Non-shallow morphology was defined as H:W ≥ 1, characterized by more vertically oriented lesions. Lesions with D:N < 2 were classified as wide-necked (red). Branching geometry was characterized based on the degree of overlap between the left and right branch point areas when projected onto the bifurcation plane. Staggered configurations exhibited < 20% overlap between the origins of the bifurcation branches (orange), whereas coplanar configurations exhibited ≥ 20% overlap between branch point areas (purple). Branch angle was measured as the angle between the extrapolated parent vessel axis and each branching daughter vessel, with black asterisks denoting aneurysms with a branch angle ≥ 90° (obtuse) between the extrapolated parent-vessel axis and the daughter branch.

All aneurysms were assessed for wide-neck morphology, defined as a neck width of 4 mm or greater or a dome-to-neck ratio less than 2. Circumferential involvement of the bifurcation was documented for each case with classification based on height-to-width ratio, dome-to-neck ratio, and branching pattern (Fig. 2).

Patient demographic, clinical, and procedural data were extracted from electronic medical records. Baseline characteristics included patient age, sex, pretreatment modified Rankin Scale (mRS) score, relevant comorbidities, and anticoagulation therapy status. Aneurysm-specific parameters included location, maximum dimension, and volumetric measurements. Aneurysm volumes were calculated using either Siemens syngo VIA aneurysm analysis software (Siemens Healthineers, Erlangen, Germany) or 3D Slicer open-source software platform (version 5.0, www.slicer.org).

The primary efficacy endpoint was aneurysm occlusion status assessed using the WEB Occlusion Scale (WOS) [23]. Aneurysm occlusion was evaluated on follow-up imaging obtained at 3 to 6‑month and 12-month intervals. Follow-up imaging modality included digital subtraction angiography (DSA), computed tomography angiography (CTA), or magnetic resonance angiography (MRA), selected based on clinical indication, patient preference, and institutional availability. DSA was preferred for its superior spatial resolution in detecting subtle in-stent stenosis and residual aneurysm filling, while non-invasive modalities were utilized when patients declined catheter angiography. Secondary outcomes included the presence and severity of in-stent stenosis on follow-up angiography. Safety outcomes included procedure-related complications, periprocedural neurological events, and delayed thromboembolic or hemorrhagic complications. Clinical outcomes were assessed using mRS scores at 6‑month and 12-month follow-up. Antiplatelet therapy regimens at each follow-up time point were also recorded.

Statistical analyses were performed using R (version 4.4.1). Continuous variables are presented as medians with interquartile ranges, and categorical variables as frequencies with percentages. Between-group comparisons were performed using the Wilcoxon rank-sum test, with *p* less than 0.05 considered statistically significant.

## Results

### Patient Cohort & Aneurysm Morphology

A total of 21 aneurysms in 21 patients were included in this study (Table 1). The median age was 71 years (IQR 64–76), and 15 patients (71.4%) were female. All aneurysms were unruptured. The majority of patients (90.4%) had a preoperative mRS score of 0. Two patients (9.5%) were receiving long-term anticoagulation therapy with apixaban for unrelated comorbidities, which was temporarily discontinued for the procedure and restarted postoperatively. Dual antiplatelet therapy regimens varied, with aspirin/clopidogrel being the most common combination in 15 patients (71.4%), followed by aspirin/ticagrelor in 4 patients (19.0%), and aspirin/prasugrel in 1 patient (4.8%). One patient was allergic to aspirin and received prasugrel monotherapy.

**Table 1:** Patient Demographics, Aneurysm Characteristics, Treatment, and Outcomes

Characteristic	Level(s)	Overall (*n* = 21)
**Baseline Patient Characteristics**		
*Age (Median (IQR)), years*	*–*	*71 (64, 76)*
Preoperative mRS (%)	0	19 (90.4)
	1	1 (4.8)
	4	1 (4.8)
Gender (%)	Female	15 (71.4)
Baseline Anticoagulation Use	Apixaban	2 (9.5)
	None	19 (90.5)
**Antiplatelet Management**		
Antiplatelet Medication (%)	ASA/ticagrelor	4 (19.0)
	ASA/prasugrel	1 (4.8)
	ASA/clopidogrel	15 (71.4)
	Prasugrel *	1 (4.8)
Preop P2Y12 (Median (IQR)), PRU	–	74.25 (48.9, 115.3)
**Aneurysm Location**		
Side (%)	Left	3 (14.3)
	Right	5 (23.8)
	Midline	13 (61.9)
Location (%)	MCA Bifurcation	7 (33.3)
	Basilar	7 (33.3)
	ACoA	6 (28.6)
	ICA Terminus	1 (4.8)
**Aneurysm Size**		
Average Height (Median (IQR)), mm		4.56 (4.1, 5.8)
Average Width (Median (IQR)), mm		4.50 (3.7, 6.6)
Average Neck (Median (IQR)), mm		3.90 (2.9, 5.7)
Aneurysm Volume (Median (IQR)), mm^3^		81.35 (46.0, 147.1)
Proximal stented Vessel (Median (IQR)), mm		2.80 (2.4, 3.0)
Distal stented Vessel (Median (IQR)), mm		1.83 (1.7, 2.3)
**Stent Technique Variables**		
Access Site (%)	Radial	17 (81.0)
	Ulnar	3 (14.3)
	Radial & Ulnar	1 (4.8)
Extent of Shouldering (%)	Minimal	3 (14.3)
	Moderate	8 (38.1)
	Maximal/Ring Sign	10 (47.6)
Parallel Selection of Stented Vessel (%)	Yes	4 (19.0)
Minimal in-stent stenosis	Yes	2 (9.5)
**3 to 6‑Month Follow-Up Outcomes (*n*** **=** **16)**		
WEB Occlusion Score* (%)	Grade A	12 (75)
	Grade AB	2 (12.5)
	Grade B	1 (6.25)
	Grade C	1 (6.25)
**6 to 12-Month Follow-Up Outcomes (*n*** **=** **11)**		
WEB Occlusion Score* (%)	Grade A	10 (90.9)
	Grade AB	1 (9.1)

*Patient with documented aspirin allergy.

*GradeA: complete occlusion, Grade AB: complete occlusion with neck recess, Grade B: Neck remnant, Grade C: Aneurysm remnant; Grade A, AB & B considered adequate occlusion

The MCA bifurcation and basilar artery were the most common aneurysm locations, each accounting for 7 cases (33.3%), followed by the anterior communicating artery in 6 cases (28.6%) and the internal carotid artery terminus in 1 case (4.8%) (Table 1). Baseline aneurysm characteristics demonstrated a median height of 4.56 mm (IQR 4.1‑5.8), width of 4.50 mm (IQR 3.7‑6.6), neck of 3.90 mm (IQR 2.9‑5.7), and volume of 81.35 mm^3^ (IQR 46.0-147.1). The median dome-to-neck ratio was 1.19 (IQR 1.09‑1.38) (Fig. S1). The median proximal stented parent vessel diameter was 2.80 mm (IQR 2.4‑3.0), and the distal branch vessel diameter was 1.83 mm (IQR 1.7‑2.3).

All 21 aneurysms demonstrated wide-neck morphology with circumferential involvement of the bifurcation, informing our decision to employ the dual-device WEVO technique rather than standalone WEB device treatment. Using our novel classification system based on 3D reformations and cross-sectional neck analysis (Fig. 2), 11 aneurysms (52.4%) were classified as pancake morphology (height-to-width ratio less than 1) and 10 aneurysms (47.6%) as non-shallow morphology (height-to-width ratio of 1 or greater) (Fig. S1). Branching geometry analysis revealed 14 coplanar configurations (66.7%) and 7 staggered configurations (33.3%), with no significant association between morphology type and branching pattern (*p* > 0.05). Most aneurysms (90.5%) arose at bifurcations with acute branching geometry (< 90 degrees), while 2 aneurysms (9.5%) demonstrated obtuse branch angles (Table 1; Fig. 2).

### Procedural Outcomes

Procedural technical variables are summarized in Table 1, Table S1, and S2. Radial arterial access was utilized in 17 patients (81.0%), with ulnar access in 3 patients (14.3%) and combined radial-ulnar access in 1 patient (4.8%). Successful stent deployment with neck scaffolding and immediate contrast stasis was achieved in all cases (100%). The median WEB device volume used was 76.97 mm^3^ (IQR 56.55-150.80) (Table S1). WEB SL devices were used in 20 cases (95.2%) and WEB SLS device in 1 case (4.8%). LVIS EVO stent sizes ranged from 3.0 to 4.0 mm in diameter and 17 to 27 mm in length (Table S1).

Four patients (19.0%) underwent parallel catheterization of the branch vessel before selection of the aneurysm with the microcatheter for WEB device placement. In the remaining patients, the side branch was selected with the stenting microcatheter following WEB device deployment. Traditional jailing of the WEB device microcatheter was never performed. Two cases required LVIS EVO stent repositioning prior to final deployment; in both instances, repositioning was necessitated by suboptimal initial stent positioning relative to the aneurysm neck due to deployment mechanics rather than sizing mismatch or branch access difficulty, and was accomplished without complication.

Post-procedural microDyna CT imaging demonstrated varying degrees of shouldering: minimal shouldering in 3 patients (14.3%), moderate shouldering in 8 patients (38.1%), and maximal shouldering characterized by the ring sign in 10 patients (47.6%) (Table 1; Fig. 1). The median WEB device diameter oversizing, defined as the difference between WEB device diameter and aneurysm width, was 1.5 mm (IQR 1.23‑1.91). The median WEB device volume-to-aneurysm volume ratio was 1.02 (IQR 0.88‑1.15), indicating near-perfect volumetric matching despite the non-geometric aneurysm morphology (Fig. S2). Neither the volume ratio nor branching pattern (coplanar versus staggered) was significantly associated with the degree of shouldering achieved (Fig. S3).

One case involved a pre-existing stent across the aneurysm neck at the time of WEB deployment. The residual aneurysm neck was extremely wide at 10.3 mm. Given the presence of a prior Neuroform Atlas stent (Stryker Neurovascular, Fremont, California, USA), we maximized superior-inferior compression to achieve adequate wall apposition. One patient (4.8%) developed visualized platelet thrombus on the stent during the procedure, which resolved following intra-arterial tirofiban administration (Table S2). Two patients (9.5%) demonstrated minimal in-stent stenosis (less than 50%) seen on microDyna CT that was not flow-limiting and did not require intervention. No procedural or immediate postoperative complications occurred. Notably, no WEB device exchanges were required in our series. The two patients who demonstrated mild in-stent stenosis, detected only on Dyna CT imaging, had WEB device volume-to-aneurysm volume ratios within the range of the overall cohort.

### Follow-Up Outcomes

Sixteen patients (76.2%) completed 3‑month to 6‑month follow-up imaging (Table 1). Adequate aneurysm occlusion (WEB Occlusion Scale Grade A or AB) was achieved in 14 patients (87.5%), with 12 patients (75%) demonstrating Grade A occlusion and 2 patients (12.5%) demonstrating Grade AB occlusion. One patient (6.3%) had Grade B occlusion and one patient (6.3%) had Grade C occlusion. One patient experienced a one-point increase in mRS score at 6 months due to new-onset non-specific headache symptoms, and one patient experienced a one point increase at 12 months due to a tremor. No other changes in mRS scores were observed. The two patients with minimal in-stent stenosis demonstrated stable findings without progression.

Eleven patients (52.4%) completed 12-month follow-up imaging (Table 1). Complete aneurysm occlusion (Grade A or AB) was observed in all patients, with 10 patients (90.9%) demonstrating Grade A occlusion and 1 patient (9.1%) demonstrating Grade AB occlusion. No new complications were recorded at 12-month follow-up. In-stent stenosis remained stable in the two affected patients without clinical sequelae.

**Fig. 3:**
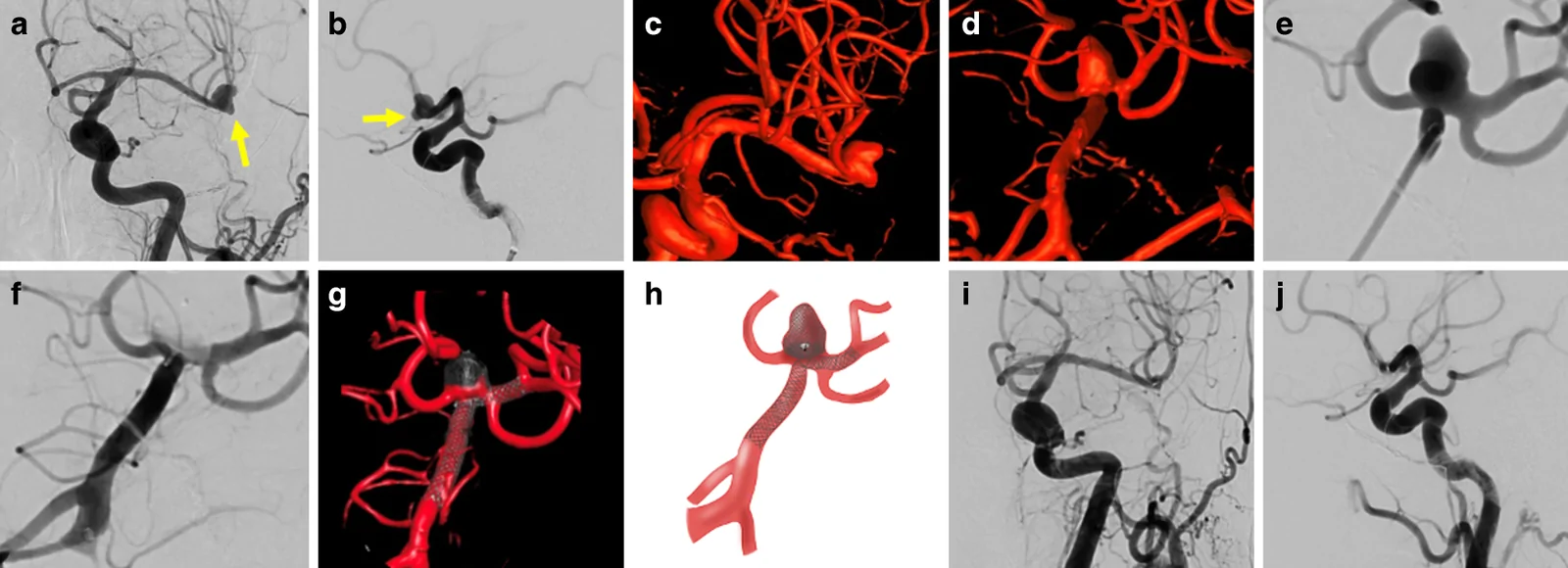
Illustrative case of a patient in their 70s presenting with headaches and found to have a non-geometric left middle cerebral artery (MCA) bifurcation aneurysm. Anteroposterior and lateral projection angiograms of the left internal carotid artery (**a**,**b**) demonstrate the aneurysm (arrows) prior to treatment. Three-dimensional digital subtraction angiography (DSA) reconstructions in lateral (**c**) and anteroposterior (**d**) views show the aneurysm projecting superiorly with two daughter blebs, measuring 6.78 × 6.00 × 4.52 mm. The aneurysm volume was 148.7 mm^3^, for which no exact WEB device match was available; therefore, an 8 × 3 mm WEB SLS was selected, providing a slightly oversized volume of 150.8 mm^3^. Following standard deployment into the aneurysm sac (**e**), the device was observed to encroach on more than 50% of the bifurcation, as anticipated. A Headway 17 microcatheter (Terumo Neuro, Aliso Viejo, California, USA) with a J-shaped guidewire was then used to navigate into the side branch, followed by deployment of a 3.5 × 23 mm LVIS EVO stent (**f**) across the aneurysm neck with moderate shouldering, achieving immediate contrast stasis. Three-dimensional post-processed DSA (**g**) and corresponding schematic illustration (**h**) demonstrate the WEB device within the aneurysm dome and the LVIS EVO stent extending across the neck into the left MCA. The patient was monitored overnight in the intensive care unit and discharged the following day on 81 mg aspirin daily without complications. At 3‑month follow-up, complete aneurysm obliteration (WEB Occlusion Scale Grade A) was observed with no in-stent stenosis or other complications, confirmed by follow-up angiography (**i**,**j**)

We present an illustrative case of a patient in their 70s with a left MCA bifurcation aneurysm (6.78 × 6.00 × 4.52 mm) treated with an 8 × 3 mm WEB SLS device and LVIS EVO stent (3.5 × 23 mm), achieving complete obliteration at 3‑month follow-up (Fig. 3).

## Discussion

In this series of 21 patients with non-geometric WNBAs, the WEVO technique achieved adequate occlusion (WOS Grade A or AB) in 87.5% of patients at 3 to 6‑month follow-up and in all cases at 12-month follow-up. Only one procedural complication occurred (4.8%), consisting of transient platelet aggregation that resolved with intra-arterial tirofiban. All patients demonstrated immediate post-procedural contrast stasis. WEVO proved safe and effective, filling a niche in treating non-geometric WNBAs in patients who are poor surgical candidates or decline microsurgical management.

This study advances the endovascular management of non-geometric WNBAs in several important ways. First, this is the first published series combining a braided stent (LVIS EVO) with an intrasaccular device (WEB device). Both WEB device and LVIS EVO are constructed from nitinol-platinum based drawn filled tubes (DFT) wire braid, [22] which allows them to work together to increase metal coverage at the aneurysm neck. Second, this is only the second published series describing clinical experience with the LVIS EVO device in the United States with long-term follow up, expanding our understanding of this braided stent platform in complex aneurysm treatment [24, 25].

### Technical Evolution and Biomechanical Rationale

Our approach evolved from early experience where stenting decisions were made intraoperatively based on immediate observation of WEB device compromise of the bifurcation or side branch. Eventually, we transitioned to pre-procedural planning based on 2D and 3D diagnostic imaging, allowing us to predetermine the need for WEVO based on aneurysm morphology characteristics before entering the angiography suite.

The WEVO technique addresses the volume-matching challenge of non-geometric aneurysms through sequential device deployment that exploits specific biomechanical properties. We first place a laterally oversized WEB device with 1.5 mm to 2 mm diameter oversizing and closest volume match while accepting potential partial herniation into the parent vessel. The WEB device’s soft, compressible nitinol structure, [2] allows safe guidewire and microcatheter passage during subsequent stent navigation without causing device migration or parent vessel injury. In 4 cases with challenging acute angle branch morphology, post-WEB device wiring was difficult, so we performed parallel catheterization with the stenting microcatheter placed in the side branch before aneurysm selection with VIA microcatheter for WEB device deployment.

We then deploy an LVIS EVO braided stent across the aneurysm neck using the shouldering technique, which involves intentional forward pressure on the delivery system that progressively packs and compresses stent cells across the neck. The shouldering technique prevented WEB device protrusion into the parent vessel in the 12 cases where the WEB volume was greater than the aneurysm volume, and elevated the compressible WEB away from the contralateral branch orifice through the compression effect while substantially increasing metal coverage at the aneurysm neck. Combined with the WEB’s intrinsic neck coverage of approximately 60%, [26] the total neck metal coverage of the WEVO construct likely reaches 70–80%.

### Comparison to Alternative Endovascular Strategies

Standalone WEB device treatment for geometric WNBAs has demonstrated adequate occlusion rates of 82–85% in large multicenter trials (WEBCAST, French Observatory, WEB-IT) [27, 28]. However, non-geometric morphology creates unreliable device-to-aneurysm volume relationships limiting treatment efficacy. Rescue stenting with laser-cut devices such as the Neuroform Atlas has been described when WEB device protrusion is encountered intraoperatively [29]. Unlike WEVO which is a deliberate strategy to expand the treatment envelope to aneurysms with suboptimal volume matching from the outset, rescue stenting addresses an intraoperative complication. Additionally, laser-cut stents provide minimal flow modification (approximately 9% [8] metal coverage versus up to 28% [22] for LVIS EVO) and lack the hemodynamic benefits and structural support that characterize the planned WEVO approach.

WEVO is not the first intrasaccular device and flow diversion combination treatment, as Abramyan et al. demonstrated that combining WEB with Pipeline Embolization Device achieved significantly superior occlusion compared to either device alone [30]. LVIS EVO’s high metal density (28%) [22] provides meaningful flow diversion at the aneurysm neck, while its braided architecture preserves branch vessel patency through the shouldering technique.

Stent-assisted coiling, typically performed with single-stent configurations (L-stent or T‑stent) or dual-stent configurations (Y-stent), constitutes the most established endovascular technique for complex WNBAs. A recent meta-analysis by Gunkan et al. demonstrated Y‑stenting to have an excellent long-term complete occlusion rate of 89% and an acceptable safety profile with intraoperative and early postoperative complication rates of 6% and 3% respectively [10]. The rate of immediate complete occlusion was 65% and was noted to improve over time due to flow diversion, [10] contrasting the immediate contrast stasis we observed in all WEVO cases. Our occlusion rates (87.5% at 3 to 6 months, 100% at 12 months) and safety profile (1 periprocedural complication, 4.8%) compare favorably with Y‑stenting, particularly given our cohort’s non-geometric morphology, though our small sample size and shorter follow-up preclude definitive conclusions about relative safety.

WEVO may further prove favorable compared to Y‑stenting due to the significant complexity of deploying two stents in addition to coils, risk of thrombotic complications with higher intraluminal metal burden, and the need for a 6F intermediate catheter especially if jailing the coiling microcatheter. WEVO, in contrast, requires only single-stent deployment, as the shouldering effect naturally elevates the compressible WEB to clear the contralateral branch orifice. This simple deployment strategy contrasts the challenging deployment of coil masses, where individual loops remain unpredictable and difficult to reliably exclude from branch vessels even with single-stent support.

Several other endovascular devices are available for the treatment of non-geometric WNBAs, each employing different techniques to achieve aneurysm occlusion. The eCLIPs device (Evasc Medical Systems Corp., Vancouver, Canada) is designed to cover the aneurysm neck, providing increased metal coverage and allows for adjunctive use with coils if desired [11]. The Contour Neurovascular System (Stryker, Fremont, California, USA) offers another intrasaccular option allowing flexible sizing but requires precise device selection for optimal results and currently lacks long-term outcome data [12]. Similarly, the SEAL device (Galaxy Therapeutics, Milpitas, California, USA) and others in development may offer improved conformability to non-geometric aneurysms or expanded size matrices that better accommodate irregular morphologies [31]. As these technologies mature and demonstrate efficacy in treating non-geometric aneurysms, the role of WEVO may diminish. Our technique should be viewed as a current solution to a specific problem rather than a definitive answer for all time.

## Limitations

Several limitations warrant acknowledgment. Our sample size of 21 patients limits statistical power and may not capture rare complications or outcomes in the broader population of patients with non-geometric WNBAs. As a single-center, predominantly single-operator retrospective series (90% of cases performed by one operator), our results reflect institutional expertise, device selection preferences, and antiplatelet regimens that may not generalize to other centers without similar experience with braided stent deployment and the shouldering technique. We lack a direct comparison group treated with alternative strategies during the same time period, preventing definitive conclusions about relative efficacy and safety. Additionally, our patient cohort primarily consisted of small aneurysms, and the technique requires validation on larger lesions. While current guidelines generally support observation for most small unruptured aneurysms, treatment decisions in our cohort were driven by morphological features associated with higher rupture risk, including irregular shape, daughter lobulations, and wide-neck bifurcation geometry, consistent with recommendations that morphological characteristics should inform management beyond size alone.

Incomplete 12-month follow-up (achieved in only 52.4% of patients) introduces potential attrition bias and may fail to identify delayed complications or late recanalization. Extended follow-up beyond 12 months will be critical to confirm occlusion stability. However, our 12-month data showing progressive improvement from 87.5% to 100% adequate occlusion is encouraging, suggesting durable thrombosis rather than device compaction.

The qualitative shouldering classification (minimal, moderate, and maximal/ring sign) introduced in this study is primarily intended as a technical teaching tool and descriptive framework to standardize deployment technique rather than a prognostic grading system. In our cohort, no significant outcome differences were observed between shouldering grades, likely reflecting both the small sample size and overall high occlusion rates across all grades. This classification provides a shared vocabulary for operators learning the technique and may facilitate comparison across future studies; however, its prognostic utility remains to be validated in larger cohorts. The shouldering technique itself requires familiarity with braided stent deployment mechanics and tactile feedback during intentional forward pressure application. We estimate a learning curve of approximately 5 to 10 cases based on the senior author’s experience to achieve consistent maximal shouldering, and formal training protocols may be needed to improve reproducibility across operators.

Regarding antiplatelet management, all patients received dual antiplatelet therapy for three months following the procedure. However, the heterogeneity in antiplatelet agents used in our cohort (clopidogrel, ticagrelor, prasugrel) is a potential confounder, as varying degrees of platelet inhibition may differentially influence thrombotic and hemorrhagic outcomes despite platelet function testing. Additionally, stent deployment at arterial bifurcations may carry higher long-term thrombotic risk compared to sidewall stenting due to altered flow dynamics and increased metal burden at branch points. Emerging data on delayed thrombotic events after braided stent placement suggest that extended single antiplatelet therapy beyond the initial dual therapy period may be warranted, and individualized duration should be considered based on patient risk factors and imaging surveillance. Prospective studies evaluating optimal antiplatelet duration in bifurcation stenting with intrasaccular devices are needed.

The WEVO construct, like other dual-device strategies, presents inherent considerations regarding retreatment flexibility. In the event of aneurysm recurrence, several options remain available. Additional coiling through the stent interstices is the most straightforward approach, leveraging the accessible interstices of the braided LVIS EVO to allow microcatheter access to the aneurysm remnant, potentially through a stent segment that is not densely packed. Alternatively, flow diversion with a Pipeline Embolization Device or an additional LVIS EVO device deployed in the parent vessel could provide further flow modification across the aneurysm neck. In refractory cases, microsurgical clipping remains a definitive option, as the intracranial construct does not preclude surgical access.

## Conclusion

The management of non-geometric WNBAs has historically required compromise between procedural feasibility and complete aneurysm occlusion. The WEVO technique addresses this treatment gap by combining intrasaccular flow disruption with deliberate neck scaffolding through planned dual-device deployment. In our 21 patient series, this approach achieved immediate contrast stasis in all cases, 100% adequate occlusion in all patients who completed 12-month follow-up, and only one minor procedural complication. While emerging intrasaccular technologies may eventually obviate the need for dual-device strategies, the WEVO technique currently expands the endovascular treatment envelope for anatomically challenging bifurcation aneurysms in patients who are poor surgical candidates and patients who prefer endovascular treatment, offering a pragmatic solution that balances safety, efficacy, and technical feasibility with existing devices.

## Supplementary Information


Supplementary tables & figures


## Data Availability

All data generated or analyzed during this study are included in this published article
