# Supplementary material for: The WEVO Technique: A Novel Dual-Device Endovascular Strategy for Non-Geometric Wide Neck Bifurcation Aneurysms
Source: Clin Neuroradiol. 2026 Mar 24;36(3):899–909. doi: 10.1007/s00062-026-01640-w (PMC13522081; doi:10.1007/s00062-026-01640-w)
Supplement: Supplementary file 1 [file 62_2026_1640_MOESM1_ESM.docx]

**SUPPLEMENTARY TABLES & FIGURES**

| **Characteristic** | **Level(s)** | **Overall (n=21)** |
| --- | --- | --- |
| Stent Size, Diameter (mm) x Length (mm) | 3 x 24 | 1 (4.8) |
|  | 3.5x17 | 3 (14.3) |
|  | 3.5x22 | 5 (23.8) |
|  | 3.5x23 | 1 (4.8) |
|  | 3x18 | 4 (19.0) |
|  | 3x24 | 1 (4.8) |
|  | 4x18 | 2 (9.5) |
|  | 4x21 | 3 (14.3) |
|  | 4x27 | 1 (4.8) |
| WEB Size, Diameter (mm) x Height (mm) | 11x6 | 1 (4.8) |
|  | 4.5x2 | 1 (4.8) |
|  | 4x2 | 2 (9.5) |
|  | 5mm SLS* | 1 (4.8) |
|  | 5x2 | 1 (4.8) |
|  | 6x2 | 5 (23.8) |
|  | 6x4 | 1 (4.8) |
|  | 7x2 | 3 (14.3) |
|  | 7x4 | 1 (4.8) |
|  | 8x3 | 3 (14.3) |
|  | 9x4 | 1 (4.8) |
|  | 9x5 | 1 (4.8) |
| Aneurysm Height (mean ± SD) mm | Anterior-posterior plane | 5.06 ± 1.96 |
|  | Lateral plane | 5.23 ± 2.09 |
| Aneurysm Width (mean ± SD) mm | Anterior-posterior plane | 5.41 ± 2.74 |
|  | Lateral plane | 5.48 ± 2.21 |
| Aneurysm Neck (mean ± SD) mm | Lateral plane | 4.17 ± 1.70 |
| Volume Of WEB Used (Median (IQR), mm^3^ |  | 76.97 (56.55, 150.80) |
| Successful Deployment (%) | Yes | 21 (100.0) |
| Single Stent (%) | Yes | 21 (100.0) |
| Guide Catheter (%) | 6F Benchmark | 2 (9.5) |
|  | 6F Sofia | 2 (9.5) |
|  | 6F Zebra | 4 (19.0) |
|  | 7F Armadillo | 3 (14.3) |
|  | 7F Zebra | 2 (9.5) |
|  | Ballast | 2 (9.5) |
|  | BMX81 | 2 (9.5) |
|  | BMX96 | 1 (4.8) |
|  | RIST6F | 3 (14.3) |
| Intermediate Catheter (%) | 5F Esperance | 3 (15.8) |
|  | 5F Sofia | 6 (31.6) |
|  | 6F Sofia | 3 (15.8) |
|  | Not Used | 2 (10.5) |
|  | Vecta 46 | 5 (26.3) |
| Microcatheter for WEB Deployment (%) | Via 17 | 14 (66.7) |
|  | Via 27 | 6 (28.6) |
|  | Via 33 | 1 (4.8) |
| Microcatheter Used to Deploy EVO (%) | Headway 17 | 15 (71.4) |
|  | Headway Duo | 6 (28.6) |
| Microwire For WEB (%) | Synchro | 11 (52.4) |
|  | Aristotle | 8 (38.1) |
|  | Balt Hybrid | 1 (4.8) |
|  | Drivewire 24 | 1 (4.8) |
| Microwire For Stent (%) | Synchro | 19 (90.5) |
|  | Aristotle | 2 (9.5) |

*5 SLS = 5 mm diameter Single Layer Sphere (only WEB configuration that is spherical; all others are cylindrical).

**Table S1. Summary of Stent Technique Variables**

| **Characteristic** | **Levels** | **Overall (n=21)** |
| --- | --- | --- |
| Stent Repositioned (%) | Yes | 2 (9.5) |
| Access site bleeding | Yes | 1 (4.8) |
| Timing of EVO Decision | Intraoperative | 6 (28.6) |
|  | Preoperative | 15 (71.4) |
| Angioplasty (%) | Yes | 2 (9.5) |
| Imaging Modality at 3/6 Month Follow-Up (%) | DSA | 12 (75) |
|  | CTA | 3 (18.8) |
|  | MRA with Contrast | 1 (6.2) |
| Imaging Modality at 12 Month Follow-Up (%) | DSA | 4 (36.4) |
|  | CTA | 2 (18.2) |
|  | MRA with Contrast | 4 (36.4) |
|  | MRI | 1 (9) |

**Table S2. Supplemental procedural outcomes and follow-up imaging characteristics**

**A.**

**B.**

**Figure S1. Aneurysm Morphology Analysis.** **(A)** The violin plot displays the distribution of dome-to-neck ratios (aneurysm width to aneurysm neck), with a median ratio of 1.19 (Q3 = 1.38, Q1 = 1.09). **(B)** The distribution of height-to-neck ratios (aneurysm height to aneurysm neck) shows a median of 1.3 (Q3 = 1.52, Q1 = 1.02), with values ranging from approximately 0 to 2.5.

**A.	B.**

**C.	D.**

**Figure S2. WEB Device and Stent Sizing Analysis. (A)** The violin plot displays the distribution of WEB device diameter to aneurysm width ratios, with a median of 1.5 (Q3 = 1.91, Q1 = 1.23). **(B)** The distribution of WEB device height to aneurysm height ratios shows a median of -2.36 (Q3 = 1.2, Q1 = -2.81). **(C)** WEB device volume to aneurysm volume ratios are shown for individual patients (n=21), with the red dashed median line at 1.02. Blue dashed lines at 0.80 and 1.10 mark the optimal oversizing range from published literature. Patient #15 is the only case with a pre-existing stent across the aneurysm neck at WEB deployment. The stent limited lateral device expansion, prompting the operator to emphasize superior-inferior compression for wall apposition, which accounts for this case's deviation from expected sizing ratios. **(D)** The distribution of stent size to maximal parent vessel diameter shows a median ratio of 0.70 (Q3 = 0.85, Q1 = 0.4).

A.

**B.**

**Figure S3. WEB Device Sizing and Aneurysm Branching Pattern Stratified by Extent of Shouldering. (A)** The box plots display the distribution of WEB device volume to aneurysm volume ratios across three categories of shouldering extent: none (n=3), medium (n=8), and maximal/ring sign (n=10). The median ratios are similar across all groups, with no significant differences detected by Kruskal-Wallis test (p=1 for none vs. medium, p=0.69 for none vs. maximal/ring sign, and p=0.47 for medium vs. maximal/ring sign). **(B)** The stacked bar chart shows the proportion of aneurysm branching patterns (coplanar in light gray, staggered in dark gray) stratified by extent of shouldering. In the none group, 100% (n=3) had coplanar branching. In the medium group, 62% (n=5) had coplanar and 38% (n=3) had staggered branching. In the maximal/ring sign group, 60% (n=6) had coplanar and 40% (n=4) had staggered branching. Fisher's exact test showed no significant association between extent of shouldering and branching pattern (p=0.6749).
